# Aberrant Brain Spontaneous Activity and Synchronization in Type 2 Diabetes Mellitus Patients: A Resting-State Functional MRI Study

**DOI:** 10.3389/fnagi.2020.00181

**Published:** 2020-06-16

**Authors:** Daihong Liu, Shanshan Duan, Ping Wei, Lihua Chen, Jian Wang, Jiuquan Zhang

**Affiliations:** ^1^Department of Medical Imaging, Chongqing University Cancer Hospital, Chongqing, China; ^2^Department of Endocrinology, Southwest Hospital, Third Military Medical University (Army Medical University), Chongqing, China; ^3^Department of Radiology, PLA 904 Hospital, Wuxi, China; ^4^Department of Radiology, Southwest Hospital, Third Military Medical University (Army Medical University), Chongqing, China

**Keywords:** type 2 diabetes mellitus, APOE-ε3 homozygotes, resting-state functional magnetic resonance imaging, amplitude of low-frequency fluctuations, functional connectivity

## Abstract

The study aimed to investigate the aberration of brain spontaneous activity and synchronization in type 2 diabetes mellitus (T2DM) patients homozygous for the apolipoprotein E (APOE)-ε3 allele. In the APOE-ε3 homozygotes, 37 T2DM patients and 37 well-matched healthy controls (HC) were included to acquire blood sample measurements, neuropsychological tests, and brain functional MRI data. The amplitude of low-frequency fluctuations (ALFF) analysis was conducted to identify the brain areas with abnormal spontaneous activity. Then, the identified brain areas were taken as seeds to compute their functional connectivity (FC) with other brain regions. The two-sample *t*-test or the *Mann–Whitney U* test were applied to reveal significant differences in acquired measurements between the two groups. The potential correlations among the three types of measurements were explored using partial correlation analysis in the T2DM group. The T2DM group had elevated glycemic levels and scored lower on the cognitive assessment but higher on the anxiety and depression tests (*p* < 0.05). The T2DM group exhibited higher ALFF in the left middle occipital gyrus, and the left middle occipital gyrus had lower FC with the left caudate nucleus and the left inferior parietal gyrus (*p* < 0.05). No significant correlations were observed. T2DM patients homozygous for the APOE-ε3 allele exhibited aberrant brain spontaneous activity and synchronization in brain regions associated with vision-related information processing, executive function, and negative emotions. The findings may update our understanding of the mechanisms of brain dysfunction in T2DM patients in a neuroimaging perspective.

## Introduction

Diabetes is an established risk factor for brain dysfunction, especially cognitive impairment, which ranges from subtle cognitive changes to dementia (Biessels and Despa, 2018). A recent study reported that the global economic burden of diabetes will increase from 1.8% (2015) to 2.2% (2030) of the global GDP (Bommer et al., 2018). Since patients with type 2 diabetes mellitus (T2DM) account for the majority of the population with diabetes (Koekkoek et al., 2015), the elucidation of the underlying neurological mechanisms of T2DM-related brain dysfunction may be a crucial part of mitigating the economic burden of diabetes. Even more importantly, it may enable early diagnosis and treatment to enhance the quality of life of patients.

Functional magnetic resonance imaging (fMRI) opens avenues to study human brain function at a timescale of seconds and a spatial scale of millimeters *in vivo* (Jiang and Zuo, 2016). Experimental investigations into the domain of T2DM-related brain dysfunction have increased to characterize the aberrant brain activity of patients, from the regional oscillations to the layout of the network (Xia et al., 2017; Liu et al., 2019; Xu et al., 2019). However, several studies have yielded inconsistent results. For instance, the amplitude of low-frequency fluctuations (ALFF), which reflects brain spontaneous activity (Zuo et al., 2010), was reported to both increase (Wang et al., 2014) and decrease (Xia et al., 2013) in the bilateral middle temporal gyrus and left fusiform gyrus. The functional connectivity (FC), which reflects brain activity synchronization (Biswal et al., 1995), was reported to both increase (Xia et al., 2015) and decrease (Cui et al., 2015) in the precuneus with independent component analysis. The discrepant results across studies may reflect genetic polymorphisms, clinical or environmental heterogeneity, and so on. We focus on apolipoprotein E (APOE) variations in the present study.

The APOE gene has three allelic variations, including ε2, ε3, and ε4. The ε2 allele of APOE is considered to be protective against Alzheimer’s disease. The ε3 allele is the most common and is often regarded as the ancestral allele. Carriers of the ε4 allele were demonstrated to have a high risk of developing Alzheimer’s disease (Farrer et al., 1997; Liu et al., 2013). Patients with amnestic mild cognitive impairment who are APOE-ε2 carriers have increased FC, but APOE-ε3 and APOE-ε4 carriers have decreased FC anchored by the amygdala (Gong et al., 2017). Structural analysis has also revealed that the APOE-ε2 carriers have a more reserved volume of the medial temporal lobe than APOE-ε3 and APOE-ε4 carriers in Alzheimer’s disease patients (Groot et al., 2018); also, APOE-ε2 carriers have more volume of hippocampal gray matter than APOE-ε3 and APOE-ε4 carriers in healthy young adults (Konishi et al., 2016). The ε4 allele is the most researched in numerous studies. For instance, APOE-ε4 carriers showed specific hippocampal functional activity in a mnemonic discrimination task (Sinha et al., 2018) and increased brain activity synchronization within the default mode network (Filippini et al., 2009; Hodgetts et al., 2019). Recent evidence shows that APOE status influences cognitive impairment in T2DM patients (Zhen et al., 2018; Tang et al., 2019). The aforementioned findings suggest that the APOE polymorphisms perturb brain functional activity and structure, which may lead to inconsistencies in resting-state fMRI results in T2DM patients.

Inspired by previous work, the present study aimed to characterize the brain abnormalities in T2DM patients without the potential influence of a mutated APOE gene. We hypothesized that aberrant brain spontaneous activity and synchronization, which were assessed with ALFF and FC, respectively, may contribute to brain dysfunction in T2DM patients. The findings may provide insight into the neurological underpinnings of T2DM-related brain dysfunction.

## Materials and Methods

### Subjects

The Medical Research Ethics Committee of the Southwest Hospital examined this cross-sectional study protocol and granted the permission of the ethics (ID: KY201864, Chongqing, China). Fifty-seven T2DM patients were recruited from the inpatients of the hospital and 70 healthy controls (HCs) were recruited from communities during December 2013 and November 2016. After being informed of the study details, all participants provided written informed consent. The participants met the inclusion criteria as follows: (1) aged from 45 years to 70 years; (2) were educated at least 6 years; and (3) were right-handed. Moreover, for T2DM patients, a diagnosis by an endocrinologist according to the criteria recommended by the World Health Organization in 1999 (Alberti and Zimmet, 1998) and a disease duration of at least 1 year were required for inclusion. Participants were excluded according to the criteria as follows: (1) a history of neurological or psychiatric diseases (major depressive disorder, dementia, multiple sclerosis, schizophrenia, amyotrophic lateral sclerosis, and epilepsy); (2) brain organic abnormalities (trauma, stroke, tumor, white matter or basal ganglia lesions rated ≥2 scores (Wahlund et al., 2001) or a history of brain surgery); (3) disabilities; and (4) contraindication to participation in MRI scans. Moreover, T2DM patients with severe complications were also excluded, including diabetic foot, retinopathy, nephropathy. The subjects were selected randomly based on ε3-homozygosity, taking into account only age, sex, body mass index (BMI) and level of education to match the two groups for these variables. Among the 57 T2DM patients, 11 ε4-carriers and four ε2-carriers were excluded according to the subsequent APOE genotyping analysis. One patient was excluded as he was 73 years old. And four subjects were excluded in the inter-group matching of age, sex, education, and BMI. Among the 70 HCs, 17 ε4-carriers and eight ε2-carriers were excluded according to the subsequent APOE genotyping analysis. Eight HCs were excluded in the inter-group matching of age, sex, education, and BMI. We thus obtained a study population of 37 patients with T2DM and 37 HCs.

### Clinical Evaluation

All subjects underwent a standardized clinical evaluation to obtain demographic and physical information and biometric measurements. First, the age, sex, handedness, years of education, and dates of T2DM diagnoses were acquired from questionnaires and interviews. Second, the physical data, including height, weight, and resting arm arterial blood pressure, were obtained on the spot. BMI was calculated according to the established formula [BMI = (weight in kg)/(height in m)^2^]. Third, medication use was obtained from medical records and interviews. Fourth, after fasting for at least 6 h, venous blood samples were collected for laboratory testing, including glycemic parameters, lipid levels, thyroid function parameters, renal function parameters, and homocysteine levels (listed in Table 1).

**Table 1:** Demographic and clinical data comparisons.

	T2DM	HC	*p*-values
Age (years)	57.57 ± 7.12	57.86 ± 5.68	0.843
Sex (male/female)	24/13	17/20	0.102^a^
Education (years)	12 (9, 14)	12 (9, 12)	0.853^b^
BMI (kg/m^2^)	25.07 ± 2.68	24.20 ± 4.09	0.283
Systolic blood pressure (mmHg)	130.11 ± 16.93	136.62 ± 16.69	0.100
Diastolic blood pressure (mmHg)	81.22 ± 10.15	80.54 ± 9.76	0.771
Disease duration (years)	8.70 ± 5.51	NA	NA
HbA_1c_ (%)	7.40 (6.65, 8.60)	5.70 (5.40, 5.95)	<0.001^*b^
Fasting plasma glucose (mmol/L)	7.74 ± 2.85	5.27 ± 0.50	<0.001*
Fasting insulin (mIU/L)	14.87 (9.61, 23.82)	11.63 (9.11, 17.39)	0.258^b^
Fasting C-peptide (ng/ml)	2.08 ± 1.11	2.10 ± 0.92	0.917
FT3 (pmol/L)	4.25 ± 0.83	4.99 ± 0.79	<0.001*
FT4 (pmol/L)	15.34 ± 2.30	16.58 ± 1.60	0.009*
TSH (mIU/L)	2.11 (1.42, 2.61)	1.96 (1.49, 3.35)	0.261^b^
Total cholesterol (mmol/L)	4.93 ± 1.20	5.22 ± 1.14	0.280
Triglyceride (mmol/L)	1.37 (1.05, 2.29)	1.12 (0.89, 1.46)	0.025^*b^
HDL cholesterol (mmol/L)	1.23 ± 0.29	1.47 ± 0.35	<0.001*
LDL cholesterol (mmol/L)	3.15 ± 0.94	3.30 ± 0.78	0.449
Blood urea nitrogen (mmol/L)	5.40 (4.25, 7.00)	5.30 (4.80, 5.95)	0.779^b^
Serum creatine (μmol/L)	64.00 (57.00, 78.00)	75.00 (63.00, 84.00)	0.070^b^
Uric acid (μmol/L)	318.76 ± 71.82	331.84 ± 68.13	0.424
Cystatin C (mg/L)	0.72 (0.65, 0.87)	0.73 (0.69, 0.87)	0.387^b^
Homocysteine (μmol/L)	10.36 (8.28, 15.08)	9.93 (8.24, 12.06)	0.364^b^

*p < 0.05. Data are presented as n for proportions, means ± SD for normally distributed continuous data, and median (QR) for non-normally distributed data. ^a^The *p*-value for sex was obtained using the χ^2^ test. ^b^The *p*-value was obtained using the *Mann–Whitney U* test. T2DM, type 2 diabetes mellitus; HC, healthy controls; BMI, body mass index; FT3, free triiodothyronine; FT4, free thyroxine; TSH, thyroid-stimulating hormone; HDL, high-density lipoprotein; LDL, low-density lipoprotein; NA, not applicable.

### APOE Genotyping

The venous blood samples of all recruited subjects were also provided for APOE genotyping for the presence of the APOE ε2, APOE ε3, and APOE ε4 alleles [Invitrogen Trading (Shanghai) Co., Limited]. The genotypic distribution for those 127 participants was ε2ε2 (0/127, 0%), ε2ε3 (12/127, 9.4%), ε2ε4 (0/127, 0%), ε3ε3 (87/127, 68.5%), ε3ε4 (28/127, 22.0%), and ε4ε4 (0/127, 0%). Because APOE-ε2 and APOE-ε4 carriers are relatively rare and ε3 is often considered to be the “wild-type” allele (Hu et al., 2011), we included only people carrying the homozygous phenotype ε3ε3.

### Neuropsychological Evaluation

Extensive neuropsychological evaluation was conducted using a battery of neuropsychological tests before an MRI scan. General cognitive screening measures included the Mini-Mental State Examination (MMSE) and the Montreal Cognitive Assessment (MoCA). Then, the following major cognitive domains were assessed: executive function and psychomotor speed (Trail Making Test, TMT), mental flexibility (Verbal Fluency Test, VFT), working memory (Digital Span Test, DST), and episodic memory (Auditory Verbal Learning Test, AVLT). Moreover, anxiety and depression were also evaluated with the Hamilton Anxiety Scale (HAMA) and the Hamilton Depression Scale (HAMD), respectively. A trained neuropsychologist blinded to the grouping situation administered the test battery. Every subject took approximately 60 min to complete all the tests. Please find the details in Table 1.

### MRI Data Acquisition Parameters

On the same day as the clinical and neuropsychological evaluation, an MRI scan was performed with a 3.0 Tesla MR scanner (Trio, Siemens healthineers, Erlangen, Germany) using a 12-channel head coil. Subjects lay supine and were instructed to stay awake with their eyes closed and relaxed during the scan. Earplugs were used to alleviate the influence of scanner noise, and foam pad were used to restrict head movement. Conventional was acquired for brain lesion detection, including T2-weighted images and fluid-attenuated inversion recovery images, as well as high-resolution T1-weighted structural images. High-resolution T1-weighted structural images and resting-state functional images were obtained for subsequent MRI data preprocessing. The parameters are the same as our previous study (Liu et al., 2018).

### MRI Data Processing

Two radiologists reviewed the T1-weighted, T2-weighted, and fluid-attenuated inversion recovery images to identify brain organic abnormalities and to score white matter changes. Both of the radiologists have at least 5 years of work experience and were blinded to the group status, and none of the participants were excluded based on the above exclusion criteria. The structural and functional images were preprocessed with the toolbox for Data Processing and Analysis for Brain Imaging (DPABI V4.0)^1^ software in a standard protocol (Yan et al., 2016). First, the original DICOM format data were converted into a NIfTI format. Then, the first 10 volumes of individual resting-state functional images were removed to avoid their probable heterogeneity. Next, slice timing was performed to correct the signal acquisition time differences between slices of the remaining 230 volumes. Realignment was performed to correct for head motion so that the brain across images was in the same position. This step automatically generated a report of head motion based on the realignment parameters, which could be the reference for subject exclusion for head motion >2.0 mm in any direction or >2.0° of any angle in Montreal Neurological Institute (MNI) coordinates. No subjects met the exclusion criteria. Then, covariate regression was conducted to remove the nuisance variables, including head motion parameters, white matter signals, and cerebrospinal fluid signals. Since global signal regression is controversial, it was not executed in the present study (Saad et al., 2012). To make inter-subject comparisons feasible, the individual functional images were spatially normalized to standard MNI space using unified segmentation on T1 images. Detrending was applied to reduce the systematic signal drift with time using a linear model. Further, the data were band-filtered (0.01–0.10 Hz) to reduce the effects of very-low-frequency and high-frequency physiological noise. Finally, the data were spatially smoothed with a 4 mm full-width half-maximum to improve the signal-to-noise ratio.

The measure ALFF was calculated based on the preprocessed images. The resulting images were standardized with mean-regression to reduce the impact of many sources of nuisance variation (Yan and Zang, 2010). Then, the correlation coefficients of time series between the seed regions and each voxel of the rest of the brain were calculated to obtain the FC pattern of every subject. It is worth noting that the brain regions with aberrant ALFF were considered as the seed regions according to the subsequent inter-group comparison of ALFF. Fisher’s *r*-to-*z* transformation was performed to improve the normality of the correlation coefficient values of FC.

### Statistical Analysis

Numeric data analyses were carried out with SPSS software (version 25.0^2^). First, the inter-group comparison of nominal variables (sex) was performed using the *Chi*-square (*χ*^2^) test. Then, the *Kolmogorov-Smirnov* test was applied in each group to verify the normality of the other numerical data distribution. According to the normality or non-normality, the two-sample *t*-test and the *Mann–Whitney U* test was applied to reveal significant differences between the T2DM group and the HC group. Values of *p* < 0.05 were considered statistically significant.

Intra- and inter-group analyses of ALFF and FC map analyses were conducted in the Statistical Analysis module of DPABI V4.0 software. First, the one-sample *t*-test was performed to check the ALFF and FC distribution pattern in each group (compared to “0”). Then, the two-sample *t-test* was performed to reveal the differences in ALFF and FC between the T2DM group and the HC group with age, sex, education years and BMI imported as covariates. The resulting maps were corrected for multiple comparisons using the Gaussian random-field theory based on the estimated effective smoothness (*p* < 0.05). As mentioned above, the brain regions with significant differences in ALFF were saved as masks to calculate the seed-based FC in each group.

The ALFF values and FC *z* scores of aberrant brain regions were extracted to investigate the relationships with neuropsychological test scores and clinical data in the T2DM group. To be specific, partial correlation analyses were conducted among the functional MRI parameters, neuropsychological performances, and diabetes-related blood measurements with adjustment for age, sex, education years, and BMI.

## Results

### Demographic and Clinical Data Comparison

According to the two-sample *t*-test, significantly elevated levels of HbA_1c_, fasting plasma glucose and triglyceride and reduced levels of free triiodothyronine (FT3), free thyroxine (FT4) and high-density lipoprotein (HDL) cholesterol were observed in the T2DM group compared to the HC group (*p* < 0.05). There were no significant differences between the T2DM group and the HC group in terms of age, sex, education years, BMI, systolic and diastolic blood pressure and other blood chemistry parameters (*p* > 0.05; Table 1). Also, the T2DM patients were undergoing standard treatment with one or more medications according to their state, including insulin, biguanides, sulfonylureas, glinides, α glucosidase inhibitors, and thiazolidinediones.

### Neuropsychological Test Comparison

According to the two-sample *t*-test, the T2DM patients scored significantly lower in the MoCA but higher in the HAMA and HAMD than the HC group (*p* < 0.05). There were no significant inter-group differences in the other neuropsychological tests, including the MMSE, TMT, VFT, DST, and AVLT (Table 2).

**Table 2:** Neuropsychological test comparison.

	T2DM	HC	*p*-values
MMSE	28.00 (27.00, 29.00)	29.00 (27.50, 29.00)	0.137^a^
MoCA	22.00 (21.00, 24.50)	24.00 (22.50, 26.00)	0.003^*a^
TMT-A	58.00 (46.00, 110.00)	56.00 (39.50, 86.50)	0.083^a^
TMT-B	145.00 (113.00, 180.50)	115.50 (100.25, 195.50)	0.425^a^
VFT	42.43 ± 6.62	40.62 ± 7.58	0.277
DST forward	9.00 (8.00, 10.00)	10.00 (8.00, 10.00)	0.170^a^
DST backward	4.00 (3.00, 4.50)	4.00 (3.00, 4.00)	0.559^a^
AVLT immediate recall	6.52 ± 1.90	6.76 ± 1.42	0.544
AVLT short-term delayed recall	7.00 (4.00, 9.50)	7.00 (6.00, 9.00)	0.719^a^
AVLT long-term delayed recall	5.00 (2.50, 8.50)	6.00 (5.00, 8.50)	0.140^a^
AVLT long-term delayed recognition	11.00 (8.00, 13.00)	11.00 (8.50, 13.50)	0.387^a^
AVLT total score	29.15 ± 11.28	31.84 ± 7.46	0.230
HAMA	7.00 (3.50, 10.5)	3.00 (2.00, 6.00)	<0.001^*a^
HAMD	5.00 (3.00, 10.00)	2.00 (1.00, 4.00)	<0.001^*a^

*p < 0.05. Data are presented as the mean ± SD for normally distributed continuous data and the median (QR) for non-normally distributed data. ^a^The *p*-value was obtained using the *Mann–Whitney U* test. MMSE, Mini-Mental State Examination; MoCA, Montreal Cognitive Assessment; TMT, Trail Making Test; DST, Digital Span Test; VFT, Verbal Fluency Test; AVLT, Auditory Verbal Learning Test; HAMA, Hamilton Anxiety Scale; HAMD, Hamilton Depression Scale.

### ALFF and FC Analysis

**Figure 1:**
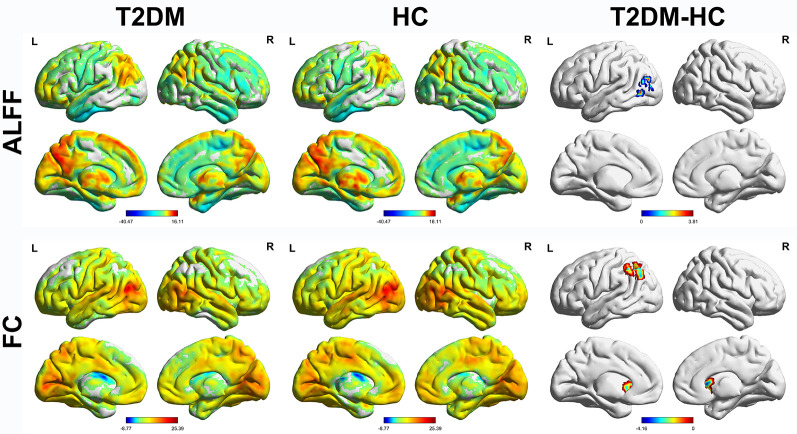
Amplitude of low-frequency fluctuations (ALFF) and functional connectivity (FC) distribution of intra-group (one-sample *t*-test) and inter-group comparisons (two-sample *t*-test). *p* < 0.05 (corrected with Gaussian random-field theory). The color bar denotes the *t*-value. R, right; L, left.

**Figure 2:**
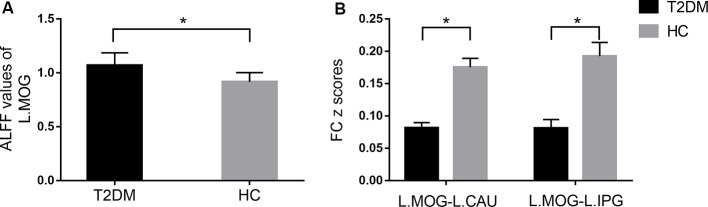
Comparison of ALFF **(A)** and FC **(B)** between the type 2 diabetes mellitus (T2DM) and healthy controls (HC) groups (two-sample *t*-test). *p < 0.05 (corrected with Gaussian random-field theory). Error bars define the SEM. R, right; L, left; MOG, middle occipital gyrus; CN, caudate nucleus; IPG, inferior parietal gyrus.

The distribution patterns of ALFF and FC in each group are shown in Figure 1 according to the results of the one-sample *t*-tests. The results of the two-sample *t*-tests show that the T2DM group exhibited significantly higher ALFF in the left middle occipital gyrus (*p* < 0.05, corrected with Gaussian random-field theory). Moreover, the left middle occipital gyrus had significantly lower FC with the left caudate nucleus and left inferior parietal gyrus in the T2DM group (*p* < 0.05, corrected with Gaussian random-field theory; Table 3, Figures 1, 2).

**Table 3:** The brain regions with aberrant amplitude of low-frequency fluctuations (ALFF) and functional connectivity (FC).

Brain regions		BA	Peak MNI coordinates			voxels	*t*-values	*p*-values
			*x*	*y*	*z*			
ALFF								
	L.MOG	37	−51	−69	0	130	3.8075	<0.05
FC								
	L.CN	11	−12	15	−3	436	−4.1624	<0.05
	L.IPG	NA	−57	−48	48	340	−3.8626	<0.05

*MNI, Montreal Neurological Institute; BA, Brodmann area; R, right; L, left; MOG, middle occipital gyrus; CN, caudate nucleus; IPG, inferior parietal gyrus; NA, not applicable. *p*-values were corrected with Gaussian random-field theory*.

### Correlation Analysis

**Figure 3:**
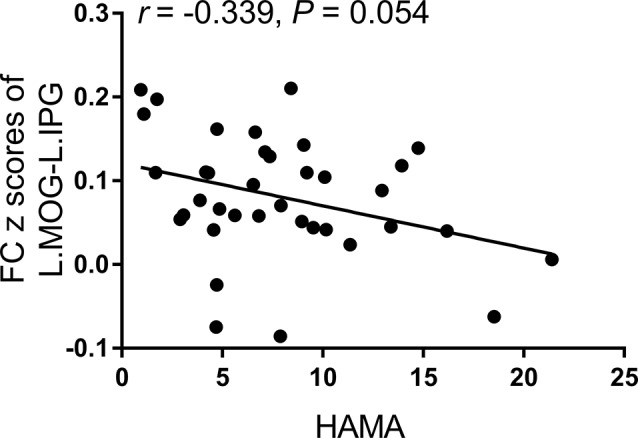
The correlation trend between the FC *z*-scores of the left middle occipital gyrus-left inferior parietal gyrus and Hamilton Anxiety Scale (HAMA) scores.

According to the partial correlation analyses, no significant correlations were observed among the aberrant functional MRI parameters, neuropsychological performances, and diabetes-related blood measurements in the T2DM group. The only correlation trend found was between the FC *z* scores of the left middle occipital gyrus-left inferior parietal gyrus and HAMA scores (*r* = −0.339, *p* = 0.054; Figure 3).

## Discussion

To better understand the function of the brain, which is considered to be the most complex system, it is essential not only to look at individual regions’ activations but also to consider how they work together through signal coherence (Bassett and Sporns, 2017). In the present study, ALFF was used to assess brain spontaneous activity and FC was used to assess synchronization between brain regions. Without the influence of APOE-ε2 and APOE-ε4, the T2DM patients exhibited aberrant ALFF in the left middle occipital gyrus, and with the left middle occipital gyrus as an anchor, changes in the FC with the left caudate nucleus and left inferior parietal gyrus were also detected. These findings may contribute to advancing the understanding of T2DM-related brain dysfunction.

Our findings identified the brain regions vulnerable to the T2DM attack without the interference of the mutated APOE gene. In previous studies, the occipital cortex, parietal cortex, and caudate nucleus exhibited abnormal functional activity both in T2DM patients and APOE-ε4 carriers (Lind et al., 2006; Peng et al., 2016; Wang et al., 2017; Liu et al., 2018). The aberrant brain regions reported in T2DM patients overlap with those in APOE-ε4 carriers, which makes it difficult to infer whether T2DM disease is a primary factor associated with functional abnormalities. It is noteworthy that the occipital cortex was reported to decrease ALFF in T2DM patients (Xia et al., 2013; Cui et al., 2014), which is contrary to our findings. However, according to the findings in APOE-ε3 homozygotes, we can infer that the functional activities of these brain regions are susceptible to T2DM disease, and the APOE gene mutation may be a candidate factor driving the aforementioned discrepancy.

Since the occipital cortex is usually considered to be the visual area, especially the primary visual cortex (Wandell, 1999), the aberrant spontaneous activity in the occipital cortex may indicate the dysfunction of vision-related information processing in T2DM patients. The visual cortex is activated in visual-processing tasks (Grill-Spector and Malach, 2004), and its gamma-aminobutyric acid levels are negatively associated with cognitive failures in daily life (Sandberg et al., 2014). In terms of T2DM, decreased cerebral blood flow in the occipital cortex was associated with worse visual-memory performance (Cui et al., 2017). In addition, T2DM patients also showed decreased regional homogeneity (Peng et al., 2016), smaller regional brain volumes (Schneider et al., 2017) and lower surface area (Bernardes et al., 2018) than HC in previous literatures.

The inferior parietal cortex is spatially adjacent to the middle occipital gyrus (Tzourio-Mazoyer et al., 2002) and is involved in executive function. According to the results of a meta-analysis, T2DM patients showed decrements in executive function (Sadanand et al., 2016). Even though the decreased ALFF of the inferior parietal lobe showed no relation with neuropsychological performance in T2DM patients (Wang et al., 2017), the reduced cerebral blood flow of the inferior parietal lobe was associated with poor performance on processing speed, which serves as a subdomain of executive function (Bangen et al., 2018). It is well known that executive function is the core component of advanced cognition (Petersen and Posner, 2012).

The caudate nucleus is involved in mood symptoms, including anxiety disorders and negative states (Amemori et al., 2018). In degenerative disorders such as Huntington’s disease and Parkinson’s disease, caudate nucleus injury was correlated with major depressive disorder (Kumar et al., 2009). Similarly, T2DM disease doubled the risk of major depression compared with the general population (Semenkovich et al., 2015). Our study also suggested that T2DM patients may be vulnerable to negative emotions according to their higher HAMA and HAMD scores. The biophysical changes in the bilateral caudate nuclei may disturb the circuits that mediate mood, especially depression (Kumar et al., 2009). In line with previous studies, the abnormality of the caudate nuclei in our findings may indicate a mood disorder in the T2DM population.

To better understand the dysfunction of the T2DM brain, it is essential to consider how the brain regions work together rather than studying them in isolation. On the one hand, high visual cortex recruitment predicted poor motor execution, which was represented by the strong activation of the inferior parietal cortex (Lebon et al., 2018). The caudate nucleus subserves not only the complex regulation of mood but also the executive function (Yang et al., 2015; Huang et al., 2016). It can excite the correct action schemas and select the appropriate sub-goals based on an evaluation of action-outcomes (Grahn et al., 2008). Therefore, the changed FC of the left middle occipital gyrus-left caudate nucleus and left middle occipital gyrus-left inferior parietal gyrus may reflect the impairment of vision-related information processing and executive function in T2DM patients. On the other hand, the inferior parietal lobe is thought to contribute to the cognitive-behavioral therapy response in major depressive disorder (Sambataro et al., 2018). The correlation trend between the decreased FC *z*-scores of the left middle occipital gyrus-left inferior parietal gyrus and the increased HAMA scores may indicate the involvement of these two brain regions in negative emotions to some extent. Also, the visual cortex was the supporting brain area crucial for facial emotion processing in major depression (Stuhrmann et al., 2011). Taking the three brain regions as a sub-network, we infer that the appearance of vision-related information processing, executive function, and mood symptoms in T2DM patients may have a combined neurological foundation.

The limitations of this study are as follows. First, because of the relatively small sample size, only T2DM patients who were APOE-ε3 homozygous carriers were enrolled in the study. The characteristic brain activity in T2DM patients who are APOE-ε2 and APOE-ε4 carriers remains to be investigated with larger sample size. Second, we preprocessed the MRI data without the removal of the global signal since the resulting negative correlations are ambiguous for interpretation (Murphy et al., 2009). Third, the two groups differed in FT4, triglyceride, and HDL cholesterol levels, even when we controlled them within normal range and attempted to achieve inter-group homogeneity of biometric measurements. Also, the different medication use in T2DM patients may affect their brain functional change patterns. The heterogeneity we cannot rule out completely may represent the real situation, but further studies are necessary to reveal their influences on brain function. Despite these limitations, the positive findings are promising and warrant further exploration.

## Conclusion

In the present study, brain spontaneous activity and synchronization were used to characterize the pattern of brain dysfunction in T2DM patients homozygous for the APOE-ε3 allele. We found that the T2DM patients exhibited aberrant brain activities in the visual cortex, caudate nucleus, and left inferior parietal gyrus, which may be associated with vision-related information processing, executive function, and negative emotions. The findings may contribute to updating the T2DM-related brain dysfunction characterization and mechanism understanding from a neuroimaging perspective. It also raises questions of the roles of APOE gene polymorphisms in cognitive impairment in T2DM patients, which should be further studied in the future.

## Data Availability Statement

All datasets generated for this study are included in the article.

## Ethics Statement

The studies involving human participants were reviewed and approved by Medical Research Ethics Committee of the Southwest Hospital (Chongqing, China). The patients/participants provided their written informed consent to participate in this study.

## Author Contributions

DL contributed to the experiments, data analysis, and the writing of the manuscript. SD, PW, and JW contributed to data collection. LC contributed to the statistical analysis. JZ are the guarantors of this study and had complete access to all data in the study. They accept responsibility for the integrity of the data and the accuracy of the data analysis.

## Conflict of Interest

The authors declare that the research was conducted in the absence of any commercial or financial relationships that could be construed as a potential conflict of interest.

## Footnotes

^1^http://rfmri.org/dpabi

^2^https://www.ibm.com/products/spss-statistics
